# Aortopexy With Plication of Kommerell Diverticulum Is an Effective Alternative to Diverticulum Resection and Reimplantation of the Aberrant Left Subclavian Artery for Surgical Management of Vascular Rings in Children

**DOI:** 10.1177/21501351251329912

**Published:** 2025-04-10

**Authors:** Adegbemisola Aregbe Perkins, Ana-Lucia Tomescu, Christopher J. Knott-Craig, Umar Boston, Thomas Spentzas, Karthik Vaidyanathan Ramakrishnan

**Affiliations:** 1 Division of Pediatric Cardiovascular Surgery, Department of Surgery, University of Tennessee Health Sciences Center and Le Bonheur Children's Hospital, Memphis, TN, USA; 2 Division of Pediatric Cardiac Surgery, Penn State Health Children's Hospital, Hershey, PA, USA; 3 Department of Pediatrics, 12310 Penn State College of Medicine, Hershey, PA, USA; 4 Department of Pediatric Critical Care Medicine, University of Tennessee Health Sciences Center and Le Bonheur Children’s Hospital, Memphis, TN, USA

**Keywords:** vascular ring, Kommerell diverticulum, aortopexy, ligamentum arteriosum

## Abstract

**Background:**

Right aortic arch with an aberrant left subclavian artery arising from a Kommerell diverticulum is the most common form of vascular ring. We report the outcomes of plication and pexy of the diverticulum in addition to division of the left-sided ligamentum to treat this lesion in children.

**Methods:**

Forty-four patients were included in the study; 22 patients underwent division of the ligamentum arteriosum alone, while the other half underwent plication and/or pexy of the Kommerell diverticulum in addition to division of the ligamentum. The primary outcome of interest was reintervention for persistent symptoms following the initial operation. The other outcome studied was symptom relief on follow-up.

**Results:**

The baseline characteristics were similar between the two groups. There was no difference in the ratio of the size of the Kommerell diverticulum to the size of the left subclavian artery between the groups (1.6 vs 1.8, *P* = .22). The incidence of reoperation was similar in both groups (5% in each group, *P* = 1); 7/22 (32%) had persistent symptoms after ligamentum division alone, while only 1/22 (5%) had persistent symptoms after plication and/or pexy (*P* = .05).

**Conclusion:**

Plication of the Kommerell diverticulum with pexy along with division of the ligamentum arteriosum is an effective alternative for treatment of right aortic arch with an aberrant left subclavian artery arising from the diverticulum.

## Background

Right aortic arch with an aberrant subclavian artery arising from a Kommerell diverticulum (KD) is the most common variant of a complete vascular ring.^1^ Surgical repair is indicated in symptomatic patients and usually consists of division of the ligamentum arteriosum. This alone leads to excellent symptom resolution in almost 75% of patients with this anatomy.^2^ Resection of the KD and reimplantation of the aberrant subclavian artery is recommended when the dimension of the KD is greater than 1.5 times the size of the left subclavian artery.^3^ Resection of the KD relieves compression on the airway/esophagus and leads to symptom resolution with a low incidence of reoperation.^3–5^ At our institution, we have followed an alternate strategy that consists of plication of the KD along with posterior aortopexy to relieve airway/esophageal compression associated with this anatomy. The aim of this study was to evaluate the outcomes of plication of KD with aortopexy in children with right aortic arch and aberrant left subclavian artery forming a vascular ring.

## Materials and Methods

This was a retrospective study approved by the Institutional Review Board with waiver of informed consent. All consecutive patients less than 18 years of age who underwent surgical repair of right aortic arch with aberrant left subclavian artery between January 2011 and December 2021 at a single center were included in the study. Data were extracted from electronic medical records. The size of the KD was measured on preoperative computed tomography (CT) as previously described by Hale and colleagues.^6^ The primary outcome was the need for reoperation for residual tracheal/bronchial compression. The secondary outcome was the persistence of symptoms without the need for reoperation and the incidence of postoperative complications.

## Operative Procedure

**Figure 1.**
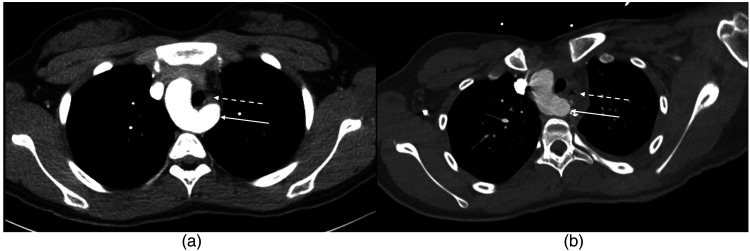
(A) Prominent Kommerell diverticulum (solid arrow) partially encircling and compressing the esophagus (broken arrow). (B) Reduced bulk of the Kommerell diverticulum (solid arrow) after plication and pexy. The diverticulum is now situated so that it no longer compresses the esophagus (broken arrow arrow).

The patient was placed in a lateral position with the left side up (Video 1). A posterior muscle-sparing thoracotomy was performed, and the pleural cavity was entered through the third or fourth intercostal space. The lung was retracted. The mediastinal pleura overlying the left subclavian artery was opened and followed down to its origin from the KD. The left-sided ductal remnant was found connected to the base of the KD. The ligamentum was dissected along its length and could be seen stringing across the esophagus. The ligamentum was divided between ligatures and this immediately opened the space around the esophagus and trachea. The KD was then plicated to reduce its bulk while preserving the origin of the left subclavian artery. The plicated diverticulum was pulled back and fixed posteriorly to the spine or head of the adjacent rib. This maneuver completely relieved compression of the esophagus. The mediastinal pleura was not reapproximated, and the chest was closed in a routine fashion with or without a chest tube in the pleural space. Postoperative CT showed the reduced bulk of the KD and the diversion of the KD away from the esophagus and toward the spine (Figure 1).

## Statistical Methods

Statistical analysis was performed using Microsoft Excel. Continuous variables are presented as median with interquartile range. Categorical variables are presented as proportions and percentages. Continuous variables were compared using the Wilcoxon rank-sum test. Categorical variables were compared using Fisher exact test.

## Results

Forty-four patients were included for analysis; 50% (n = 22) underwent division of the ligamentum arteriosum alone and the remaining 50% had KD plication and/or KD pexy. In the latter group, 7 patients had KD plication alone, 4 patients had KD pexy alone, and 11 patients had both KD plication and pexy. The preoperative characteristics between the two groups were comparable (Table 1). The KD dimension and the ratio between the dimension of the KD and the aberrant left subclavian artery were similar between the two groups; 31 (14 in the division-only group and 17 in the pexy/plication group; *P* = .32) patients had predominantly respiratory symptoms, while 13 (eight in the division group and five in the plication/pexy group; *P* = .32) had predominantly gastrointestinal symptoms. Stridor or dysphagia was seen in eight patients (two in the division-only group and six in the plication/pexy group; *P* = .18). Other symptoms included frequent respiratory tract infections, wheezing, chest discomfort, and frequent spit-ups. Two patients underwent reoperation, one in each group. One patient underwent repeat thoracotomy, adhesiolysis, and aortopexy seven years after initial ligamentum division and KD pexy. The patient was nine years old at the time of reoperation and had persistent respiratory problems with imaging evidence of airway narrowing at the site of the vascular ring. Her symptoms resolved largely after reoperation. The other reoperation was in a 26-year-old, 12 years after the initial division-only operation. He had dysphagia and underwent resection of the KD and division of the aberrant left subclavian artery with complete resolution of symptoms. On last follow-up, eight of 44 patients (18%) had persistent symptoms, the majority (7) of which were in the division-only group. The symptoms were respiratory in all patients with a predominance of wheezing and recurrent respiratory tract infections. None of the patients had imaging evidence of significant persistent airway obstruction and were managed without reoperation.

**Table 1.** Patient Characteristics and Outcome Data.^a^

	Total n = 44	Division n = 22	Division + plication/aortopexy n = 22	*P* value
Age, years	3 (0.8-8)	3 (0.8-4)	4.5 (0.75-12)	.18
Male, n (%)	27 (61%)	12(55%)	15(68%)	.54
Syndromic/chromosomal abnormalities, n (%)	6(13%)	2(9%)	4(18%)	.31
KD diameter (mm)	15.3(10.2-21.8)	10.7(7.9-11.9)	17.6(13.3-23.2)	.16
KD/LSCA	1.7	1.6	1.8	.22
Postoperative complications, n (%)	1 (2%)	1(5%)	0	1
Hospital stay, days	2 (2-3)	2(2-3.5)	2(2-3)	1
Follow-up duration (years)	3.2(1.6-7.45)	3.8(2-5.1)	3(0.5-4)	.46
Persistent symptoms, n (%)	8(18%)	7(32%)	1(5%)	.05
Reoperation, n (%)	2(5%)	1(5%)	1(5%)	1

Abbreviations: KD, Kommerell diverticulum; LSCA, left subclavian artery.

^a^ Continuous variables are expressed as medians (interquartile range). Wilcoxon rank sum test was used to compare continuous variables between the two groups. Categorical variables are expressed as number (percentage). Fisher's exact was used to compare categorical variables between the two groups.

## Comment

The findings of our study show that KD plication and/or pexy results in better symptom resolution than division of the ligamentum alone. The incidence of complications and reoperation with KD plication and/or pexy is low. Division of the ligamentum arteriosum alone is not sufficient to relieve all symptoms and hence is not recommended as a stand-alone procedure for vascular rings. Although resection of the KD and reimplantation of the left subclavian artery is recommended for KD/LSCA greater than 1.5, this criterion is not universally followed. The reasons for not resecting the KD and reimplanting the aberrant left subclavian artery can be multifactorial. Resection of KD and reimplantation of the aberrant left subclavian artery can be a challenging procedure in small children. One of the main drawbacks of resection and reimplantation is that for inexperienced surgeons who do not perform this procedure quite as often, it can be quite difficult. The left carotid artery runs between the vagus and phrenic nerves and can be difficult to find in some patients, especially for someone not familiar with this operation. This places both the vagus and phrenic nerve at risk for injury.^4^ The thoracic duct also runs in this region and can be at risk.^4,7^ Clamping the carotid artery for reimplantation can compromise blood supply to the brain in the rare instance that the circle of Willis is incomplete. In addition, the long-term patency of the reimplanted subclavian artery is not known. And there is also the theoretical risk of creating an aortic coarctation while oversewing the KD. More and more vascular rings are being diagnosed in-utero resulting in an earlier age of referral for surgical repair.^1,8^ In some of these patients, the symptoms are nonspecific and are not the classical obstructive symptoms that are seen in severe vascular rings.^8^ Although some groups have reported excellent results with primary translocation of the aberrant vascular ring,^4,9^ the results have not always been reproducible.^7^ In a reasonably large single-center experience, there was a 45% incidence of persistent symptoms after KD resection and reimplantation of the aberrant left subclavian artery.^7^ In another single-center experience, symptom relief was excellent in patients who underwent ligamentum division with pexy of the KD.^10^ Our results clearly indicate that plication and pexy of the KD can be performed with a low incidence of complications and good relief of symptoms. It is a less complex operation than reimplanting the subclavian artery, especially in small children. Division of the ligamentum arteriosum alone should not be performed as there is a higher incidence of residual symptoms with this approach.^11^

Our pragmatic approach for these patients with vascular rings is to consider the symptoms and findings on cross-sectional imaging. We reserve resection and reimplantation for the very rare case where the KD is massive, and there is a significant narrowing of the airway or esophagus with clear corresponding symptoms. This will be evident in the operating room. Once the KD has been plicated it will no longer overhang and compress the esophagus. In situations where the KD still compresses the esophagus even after plication, this would be an indication to resect the KD and reimplant the left subclavian artery. We did not find any instance where resection of the KD had to be performed in our series at the time of the primary operation. In most patients who are now referred during infancy with nonobstructive or vague respiratory or gastrointestinal symptoms, we prefer to perform plication and pexy of the KD in addition to division of the ligamentum arteriosum. Reducing the bulk of the KD in addition to situating it so that it no longer compresses the esophagus by performing a pexy results in a durable repair with good resolution of symptoms and a low incidence of reoperation for persistent symptoms. We do not recommend division of the ligament alone, as KD plication with pexy adds little to the complexity of the procedure and is associated with improved resolution of the symptoms.

## Limitations

Our study is limited by the absence of perioperative bronchoscopy and/or esophagogram in all patients to truly delineate the efficacy of our procedure. Further, we do not have follow-up images of the KD to assess its size on follow-up. The sample size is small and is insufficiently powered to make definite conclusions. Further, follow-up duration is also not very long. Some patients with KDs may present with aneurysmal dilation after decades. Our patients have not been followed-up into adulthood and the fate of the plicated KDs in our patients remains to be seen. We also acknowledge that the follow-up of these patients is often incomplete due to the heterogeneous nature of the referring providers (pulmonologists, primary care physicians, otorhinolaryngologists, etc). Documentation of symptom relief can often be vague or incomplete and that might potentially confound the results presented here. Dynamic CT may reveal more subtle obstruction of the airway in those with residual symptoms, and the absence of dynamic imaging is a limiting factor in attributing symptoms to a residual vascular ring following division of the ring alone.

## Conclusions

Plication of KD with posterior pexy along with ligamentum division is an effective alternative treatment for children with right aortic arch and aberrant left subclavian artery with a low risk of complications or reoperation.

## Supplemental Material

**Figure:** Video 1.
